# Microglial activation in the frontal cortex predicts cognitive decline in frontotemporal dementia

**DOI:** 10.1093/brain/awad078

**Published:** 2023-03-08

**Authors:** Maura Malpetti, Thomas E Cope, Duncan Street, P Simon Jones, Frank H Hezemans, Elijah Mak, Kamen A Tsvetanov, Timothy Rittman, W Richard Bevan-Jones, Karalyn Patterson, Luca Passamonti, Tim D Fryer, Young T Hong, Franklin I Aigbirhio, John T O’Brien, James B Rowe

**Affiliations:** Department of Clinical Neurosciences and Cambridge University Hospitals NHS Trust, University of Cambridge, Cambridge CB2 0SZ, UK; Department of Clinical Neurosciences and Cambridge University Hospitals NHS Trust, University of Cambridge, Cambridge CB2 0SZ, UK; Medical Research Council Cognition and Brain Sciences Unit, Cambridge CB2 7EF, UK; Department of Clinical Neurosciences and Cambridge University Hospitals NHS Trust, University of Cambridge, Cambridge CB2 0SZ, UK; Department of Clinical Neurosciences and Cambridge University Hospitals NHS Trust, University of Cambridge, Cambridge CB2 0SZ, UK; Department of Clinical Neurosciences and Cambridge University Hospitals NHS Trust, University of Cambridge, Cambridge CB2 0SZ, UK; Medical Research Council Cognition and Brain Sciences Unit, Cambridge CB2 7EF, UK; Department of Psychiatry, University of Cambridge, Cambridge CB2 0QQ, UK; Department of Clinical Neurosciences and Cambridge University Hospitals NHS Trust, University of Cambridge, Cambridge CB2 0SZ, UK; Department of Clinical Neurosciences and Cambridge University Hospitals NHS Trust, University of Cambridge, Cambridge CB2 0SZ, UK; Department of Psychiatry, University of Cambridge, Cambridge CB2 0QQ, UK; Department of Clinical Neurosciences and Cambridge University Hospitals NHS Trust, University of Cambridge, Cambridge CB2 0SZ, UK; Medical Research Council Cognition and Brain Sciences Unit, Cambridge CB2 7EF, UK; Department of Clinical Neurosciences and Cambridge University Hospitals NHS Trust, University of Cambridge, Cambridge CB2 0SZ, UK; Department of Clinical Neurosciences and Cambridge University Hospitals NHS Trust, University of Cambridge, Cambridge CB2 0SZ, UK; Wolfson Brain Imaging Centre, University of Cambridge, Cambridge CB2 0QQ, UK; Department of Clinical Neurosciences and Cambridge University Hospitals NHS Trust, University of Cambridge, Cambridge CB2 0SZ, UK; Wolfson Brain Imaging Centre, University of Cambridge, Cambridge CB2 0QQ, UK; Department of Clinical Neurosciences and Cambridge University Hospitals NHS Trust, University of Cambridge, Cambridge CB2 0SZ, UK; Wolfson Brain Imaging Centre, University of Cambridge, Cambridge CB2 0QQ, UK; Department of Psychiatry, University of Cambridge, Cambridge CB2 0QQ, UK; Department of Clinical Neurosciences and Cambridge University Hospitals NHS Trust, University of Cambridge, Cambridge CB2 0SZ, UK; Medical Research Council Cognition and Brain Sciences Unit, Cambridge CB2 7EF, UK

**Keywords:** microglial activation, PET, frontotemporal dementia, prognosis

## Abstract

Frontotemporal dementia is clinically and neuropathologically heterogeneous, but neuroinflammation, atrophy and cognitive impairment occur in all of its principal syndromes. Across the clinical spectrum of frontotemporal dementia, we assess the predictive value of *in vivo* neuroimaging measures of microglial activation and grey-matter volume on the rate of future cognitive decline. We hypothesized that inflammation is detrimental to cognitive performance, in addition to the effect of atrophy. Thirty patients with a clinical diagnosis of frontotemporal dementia underwent a baseline multimodal imaging assessment, including [^11^C]PK11195 PET to index microglial activation and structural MRI to quantify grey-matter volume. Ten people had behavioural variant frontotemporal dementia, 10 had the semantic variant of primary progressive aphasia and 10 had the non-fluent agrammatic variant of primary progressive aphasia. Cognition was assessed at baseline and longitudinally with the revised Addenbrooke's Cognitive Examination, at an average of 7-month intervals (for an average of ∼2 years, up to ∼5 years). Regional [^11^C]PK11195 binding potential and grey-matter volume were determined, and these were averaged within four hypothesis-driven regions of interest: bilateral frontal and temporal lobes. Linear mixed-effect models were applied to the longitudinal cognitive test scores, with [^11^C]PK11195 binding potentials and grey-matter volumes as predictors of cognitive performance, with age, education and baseline cognitive performance as covariates. Faster cognitive decline was associated with reduced baseline grey-matter volume and increased microglial activation in frontal regions, bilaterally. In frontal regions, microglial activation and grey-matter volume were negatively correlated, but provided independent information, with inflammation the stronger predictor of the rate of cognitive decline. When clinical diagnosis was included as a factor in the models, a significant predictive effect was found for [^11^C]PK11195 BP_ND_ in the left frontal lobe (−0.70, *P* = 0.01), but not for grey-matter volumes (*P* > 0.05), suggesting that inflammation severity in this region relates to cognitive decline regardless of clinical variant. The main results were validated by two-step prediction frequentist and Bayesian estimation of correlations, showing significant associations between the estimated rate of cognitive change (slope) and baseline microglial activation in the frontal lobe. These findings support preclinical models in which neuroinflammation (by microglial activation) accelerates the neurodegenerative disease trajectory. We highlight the potential for immunomodulatory treatment strategies in frontotemporal dementia, in which measures of microglial activation may also improve stratification for clinical trials.

## Teaser

Malpetti *et al.* report that inflammation in frontal regions is associated with faster cognitive decline in people with a clinical diagnosis of frontotemporal dementia (FTD), providing complementary information to measures of grey-matter volume. The findings highlight the potential for immunomodulatory treatment strategies in FTD.

## Introduction

Frontotemporal dementia (FTD) encompasses a pathologically and clinically heterogeneous spectrum of neurodegenerative conditions, including the behavioural variant (bvFTD),^1^ as well as the non-fluent and semantic variants of primary progressive aphasia (nfvPPA and svPPA),^2^ and right temporal variant FTD.^3^ Despite genetic, molecular and clinical differences, there is evidence for increased neuroinflammation across the whole spectrum of FTD, including activated microglia and inflammatory cytokine expression,^4,5^ post-mortem human studies of microglial dysfunction,^6–8^ convergent genome wide association studies^9–11^ and animal models.^12,13^ Despite the evidence that neuroinflammation is a central neuropathological feature of FTD, it is not clear whether it accelerates clinical progression.

A few studies have used PET to quantify microglial activation *in vivo* in FTD, building on a much larger body of PET evidence of microglial activation in Alzheimer's disease. PET ligands for the translocator protein (TSPO), such as [^11^C]PK11195, have revealed increased microglial activation in frontotemporal regions in patients with both sporadic and genetic FTD.^14–18^ [^11^C]PK11195 PET indicates differential regional activation of microglia according to clinical phenotype^14,15^ and genetic variant.^17^ The PET studies are complemented by post-mortem evidence of elevated microglial activation in frontal and temporal regions.^6,7,15^

MRI is widely used to measure structural brain changes in FTD, in cross-sectional and longitudinal studies.^19,20^ MRI indices of atrophy correlate with clinical manifestations in cross-sectional cohorts,^21–23^ whereas longitudinal volumetric changes track disease progression in each of the principal phenotypes.^24–30^ In addition, the rate of atrophy may be useful in monitoring and evaluating experimental therapies.^31^ However, the prognostic value of atrophy measures in combination with inflammation markers has not been tested in FTD.

A prognostic role of baseline inflammation would be of particular interest, because of the potential for immunomodulatory therapies to slow disease progression. This study therefore took a longitudinal, multimodal approach. Instead of simply assessing baseline and longitudinal change in a single measure, here we test whether baseline microglial activation and atrophy can predict future clinical progression. We focus on cognitive deficits that are affected across FTD syndromes, with low linguistic dependency. We test the hypothesis that higher inflammation levels and lower grey-matter volumes in the frontal and temporal lobes at baseline are independently associated with faster decline in cognitive performance.

## Materials and methods

### Participants

Thirty patients with FTD were recruited as part of the NIMROD study (Neuroimaging of Inflammation in Memory and Related Other Disorders)^32^ from the Cambridge Centre for Frontotemporal Dementia and Related Disorders’ specialist clinics. Ten patients met diagnostic criteria for bvFTD,^1^ 10 cases for nfvPPA and 10 for svPPA.^2^ Seven patients were positive carriers for pathogenic mutations associated with FTD. Baseline [^11^C]PK11195 data of a subset of these participants have been published previously;^15,17^ here we present the longitudinal cognitive follow-up. Two cases had mutations in the microtubule associated protein tau (MAPT), two in progranulin and three had hexanucleotide expansions in C9 open reading frame 72 (C9orf72).

At baseline, patients underwent 3 T MRI and [^11^C]PK11195 PET, and a standard battery of cognitive tests, which included the revised version of Addenbrooke's Cognitive Examination (ACE-R).^33^ Clinical follow-up and ACE-R were repeated at an average of 7-month intervals [mean ± standard deviation (SD) of 7.2 ± 2.6 months], up to a maximum of 57 months (4.75 years, on average 18.7 ± 13.9 months) after baseline visit.

Written informed consent was obtained from the participants. The NIMROD protocol was approved by the National Research Ethics Service's East of England Cambridge Central Committee, and the UK Administration of Radioactive Substances Advisory Committee.

### Imaging data acquisition and preprocessing

Structural MRI and [^11^C]PK11195 PET data were acquired and processed using previously described methods.^15,32^ Briefly, patients underwent a 3 T MRI scan, followed by a dynamic [^11^C]PK11195 PET scan for 75 min. MRI used Siemens Magnetom Tim Trio and Verio scanners (Siemens Healthineers) with an MPRAGE T_1_-weighted sequence, while PET used GE Advance and GE Discovery 690 PET/CT (GE Healthcare) scanners. Time intervals between baseline clinical assessment and imaging were [median (mean ± SD)]: 1.5 (1.7 ± 1.8) months for MRI, and 4.0 (4.4 ± 3.0) months for [^11^C]PK11195 PET.

Each T_1_ image was non-rigidly registered to the ICBM2009a template brain using ANTS (http://www.picsl.upenn.edu/ANTS/) and the inverse transform was applied to the Hammers atlas (resliced from MNI152 to ICBM2009a space) to bring the regions of interest to subject MRI space. The T_1_-weighted images were segmented into grey matter, white matter and CSF with SPM12 and used to determine regional grey matter, white matter and CSF volumes, and to calculate the total intracranial volume (grey matter + white matter + CSF) in each participant.

For each participant, the aligned dynamic PET image series for each scan was rigidly co-registered to the T_1_-weighted MRI image. Grey-matter volumes and [11C]PK11195 non-displaceable binding potential (BP_ND_) for each tracer were calculated in 83 cortical and subcortical regions of interest using a modified version of the Hammers atlas.^34,35^ Before kinetic modelling, regional PET data were corrected for partial volume effects from CSF. Supervised cluster analysis was used to determine the reference tissue time–activity curve for [^11^C]PK11195 and BP_ND_ values were calculated in each region of interest using a simplified reference tissue model with vascular binding correction.^36^

Modality-specific regional values were combined to obtain values for grey-matter volume, corrected for total intracranial volume and volume-weighted mean values for [^11^C]PK11195 BP_ND_ in four regions of interest: left and right frontal and temporal lobes. These were selected on the basis of previous imaging evidence^19,20^ about shared neural correlates across all clinical syndromes of FTD, and to mirror post-mortem studies on inflammation in frontotemporal regions of patients with these conditions.^7^ We ran exploratory analyses with modality-specific regional values in left and right parietal and occipital lobes, to confirm regional specificity of our hypothesis. Regional *Z*-scores were calculated for each patient using group-level region- and modality-specific means and standard deviations. Means and standard deviations were calculated from the whole cohort of patients.

### Statistical analyses

Statistical analyses used R v.4.0.0 (R Core Team) for frequentist methods and Bayesian rank-based hypothesis testing.

Age, years of education, baseline cognitive and clinical scores were compared between the three groups with analysis of variance (ANOVA) tests, whereas sex was compared with the Chi-square test (see Table 1). Patients with nfvPPA were older than bvFTD and svPPA (after Bonferroni correction of *post hoc* contrasts). ANOVAs were also performed in each region to compare modality-specific regional *Z*-scores across diagnostic groups (Supplementary Table 1).

**Table 1:** Demographic and clinical characteristics for patient groups

	All patients	bvFTD	nfvPPA	svPPA	Group effect	*Post hoc* comparisons (Bonferroni)
*n*	30	10	10	10	-	-
Sex (F/M)	14/16	5/5	7/3	2/8	NS	-
Age (years; mean ± SD)	66.1 ± 8.7	60.0 ± 8.6	71.1 ± 8.8	67.2 ± 4.7	*F*(2,27) = 5.5, *P* = 0.010*	bvFTD < nfvPPA
Education (years; mean ± SD)	13.0 ± 2.9	12.4 ± 2.8	11.5 ± 2.0	15.2 ± 2.5	*F*(2,27) = 6.2, *P* = 0.006*	bvFTD < svPPA and nfvPPA < svPPA
Genetic cases	2 MAPT, 2 GRN, 3 C9orf72	2 MAPT, 1 GRN, 3 C9orf72	1 GRN	-	-	-
Symptom duration (years; mean ± SD)	4.7 ± 2.4	5.3 ± 2.4	3.7 ± 2.1	5.1 ± 2.6	NS	-
Survival rate within 5 years (deceased/alive)	11/19	3/7	6/4	2/8	NS	-
CDR sum of boxes (mean ± SD)	8.2 ± 5.5	13.4 ± 5.0	4.3 ± 3.5	7.1 ± 3.3	*F*(2,27) = 13.68, *P* = 0.001*	nfvPPA < bvFTD and svPPA < bvFTD
ACE-R (/100—mean ± SD)	66.2 ± 17.8	54.7 ± 17.9	77.9 ± 15.6	65.9 ± 12.6	*F*(2,27) = 5.61, *P* = 0.009*	bvFTD < nfvPPA
Att/ori ACE-R (/18—mean ± SD)	15.1 ± 3.7	12.1 ± 4.4	15.9 ± 2.8	17.3 ± 1.1	*F*(2,27) = 7.64, *P* = 0.002*	bvFTD < nfvPPA and bvFTD < svPPA
Fluency ACE-R (/14—mean ± SD)	4.6 ± 3.5	2.3 ± 2.2	5.7 ± 3.9	5.7 ± 3.2	*F*(2,27) = 3.85, *P* = 0.034*	NS
Lang ACE-R (/26—mean ± SD)	17.2 ± 6.0	15.9 ± 6.1	21.5 ± 4.3	14.2 ± 5.2	*F*(2,27) = 5.32, *P* = 0.011*	svPPA < nfvPPA
Memory ACE-R (/26—mean ± SD)	15.0 ± 7.5	10.7 ± 7.5	21.4 ± 6.0	13.0 ± 4.4	*F*(2,27) = 8.46, *P* = 0.001*	bvFTD < nfvPPA
Visuosp ACE-R (/16—mean ± SD)	14.3 ± 2.1	13.7 ± 2.1	13.4 ± 2.4	15.7 ± 0.5	*F*(2,27) = 4.56, *P* = 0.020*	nfvPPA < svPPA
Follow-up intervals (months, mean ± SD)	7.2 ± 2.6	6.4 ± 3.1	6.3 ± 1.1	8.8 ± 2.4	NS	-

GRN = progranulin; Att/ori = attention/orientation; F = Female; M = Male; NS = non-significant.

*Significant subgroup effect on variance, by one-way ANOVA.

To investigate cognitive changes over time, linear mixed-effects models were applied to the longitudinal ACE-R subscores for attention/orientation. We chose this subscore as a marker of cognitive function as it provides a suitable estimate of frontal function while being less influenced by language impairment than the total score.^33^ The model included the estimation of a random intercept and a random slope, accounting for individual patient differences, with time (in years) for each visit from baseline as fixed effect and cognitive scores as dependent variable. *P*-values were obtained via Satterthwaite's degrees of freedom method and via likelihood ratio tests of the model with the time effect against the null model without the time effect. The linear mixed-effects analysis was performed using the lme4 and lmerTest R packages.^37^

Associations between [^11^C]PK11195 BP_ND_ and grey-matter volume across all patients were tested including lobar values (frontal and temporal) and inter-modality Spearman's correlations. Hypothesizing a detrimental role of inflammation in FTD, we predicted negative associations between [^11^C]PK11195 BP_ND_ and grey-matter volume. Inferred *P*-values are reported both uncorrected (p) and with correction for multiple comparisons (using the false discovery rate, p-FDR).

To test the predictive value of [^11^C]PK11195 BP_ND_ and grey-matter volume on cognitive decline at the group level, both one- and two-step prediction approaches were applied. Across all approaches, we tested for positive associations between grey-matter volumes and cognitive performance over time, and negative associations between microglial activation levels (quantified using [^11^C]PK11195 BP_ND_) and cognitive performance over time.

#### One-step prediction approach

Linear mixed-effects models used longitudinal cognitive scores as the dependent variable, with imaging modality-specific values at baseline and time interval for each visit from baseline (years) as predictors. A random intercept was included to account for variability between subjects. Modality-specific left and right frontal and temporal lobar values were mean-centred and scaled across all patients (*Z*-scores), and included in the model as predictors, alongside age at the baseline visit, years of education and baseline cognitive performance as covariates of no interest. Inferred *P*-values are reported both uncorrected (p) and with correction for multiple comparisons (p-FDR).

We then ran similar models adding the interaction term between time and diagnosis, to take into account the regional anatomical variance of the three main syndromes, which could lead to varying regional associations between the brain changes and outcome. We ran similar models including a variable for the genetic status (1 = gene mutation, 0 = sporadic/unknown gene cases) and its interaction with time to test whether this plays a role in the main results. Further exploratory analyses were run using other cognitive domains estimated from ACE-R (i.e. memory, language, fluency, visuospatial domains) as outcome variables.

#### Two-step prediction approach

The one-step procedure brings estimation challenges with a modest sample size. Therefore, we confirmed our main findings with a simpler two-step prediction procedure. First, we extracted individual slope values from the initial linear mixed-effects model on longitudinal cognitive scores (with only time as a fixed effect), which represent the individual rate of cognitive decline. Second, we included these estimated cognitive slopes in correlation analyses with modality-specific imaging values. Specifically, negative associations of cognitive performance over time with microglial activation (i.e. [^11^C]PK11195 BP_ND_) and positive associations with grey-matter volumes were tested. We applied both frequentist and Bayesian Spearman's correlation analyses (https://osf.io/gny35/) to ensure inferential robustness, allowing us to quantify evidence in favour of the null hypothesis (of no predictive value).

### Data availability

Anonymized data may be shared on request to the corresponding or senior author from a qualified investigator for non-commercial use, subject to restrictions according to participant consent and data protection legislation.

## Results

**Figure 1:**
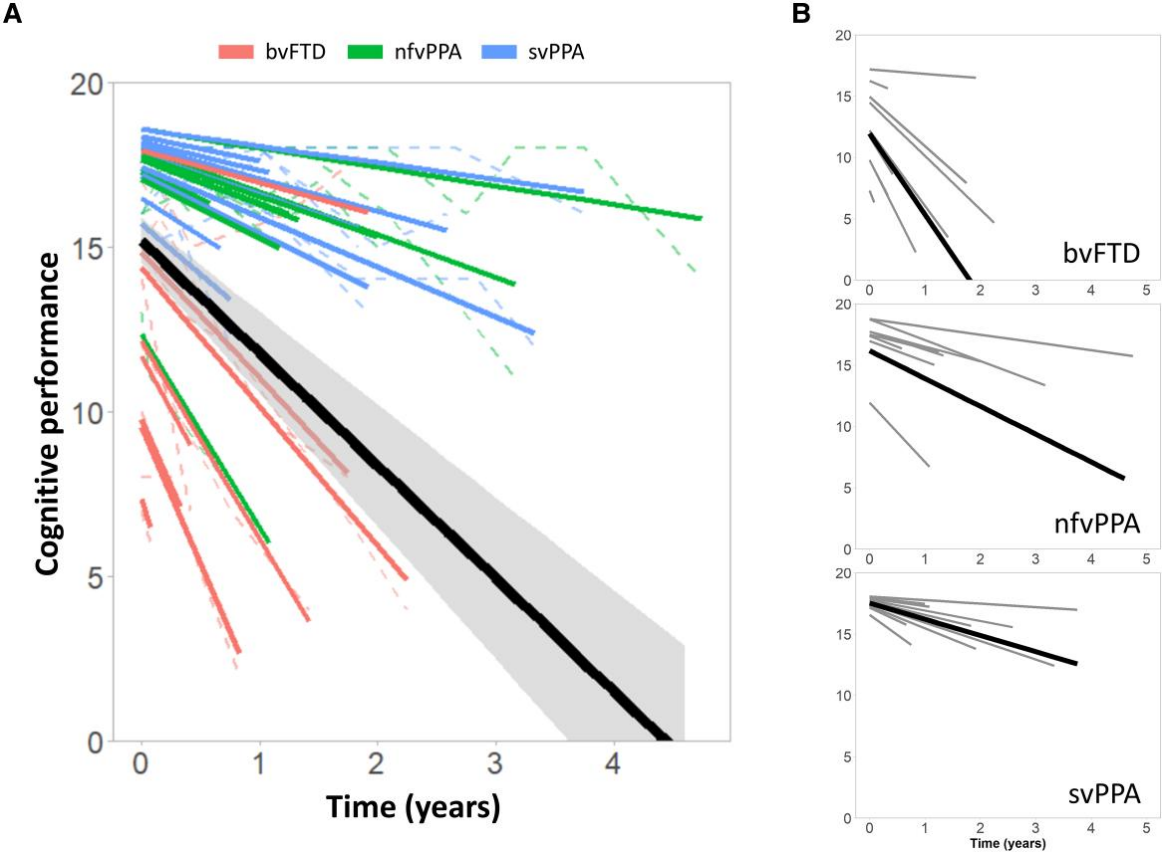
**Cognitive decline over time**. Cognitive performance measured by attention/orientation subscore of the ACE-R. (**A**) Cognitive decline across all patients. Dashed lines represent raw scores, bold lines chart the time-course for individual patients estimated by the linear mixed-effects model, whereas the thick line represents the linear estimated change at the group level. (**B**) Cognitive decline in clinical subgroups. Bold lines represent individual trajectories, while the thick line maps the linear estimated change for each group.

Time had a strong effect on ACE-R attention scores at the group level (Δ*χ*^2^ = 24.89, Δd.f. = 1, *P* < 0.0001), with ACE-R attention score declining by an average 3.42 points per year (see Supplementary Table 2 for standardized effect size and power calculations for clinical trials using this score as an outcome measure). However, there was considerable between-subject variability (random effects—Δ*χ*^2^ = 45.26, Δd.f. = 2, *P* < 0.0001; Fig. 1). See Supplementary Fig. 1 for changes over time in other ACE-R subscores (fluency, language, memory, visuospatial domains).

**Figure 2:**
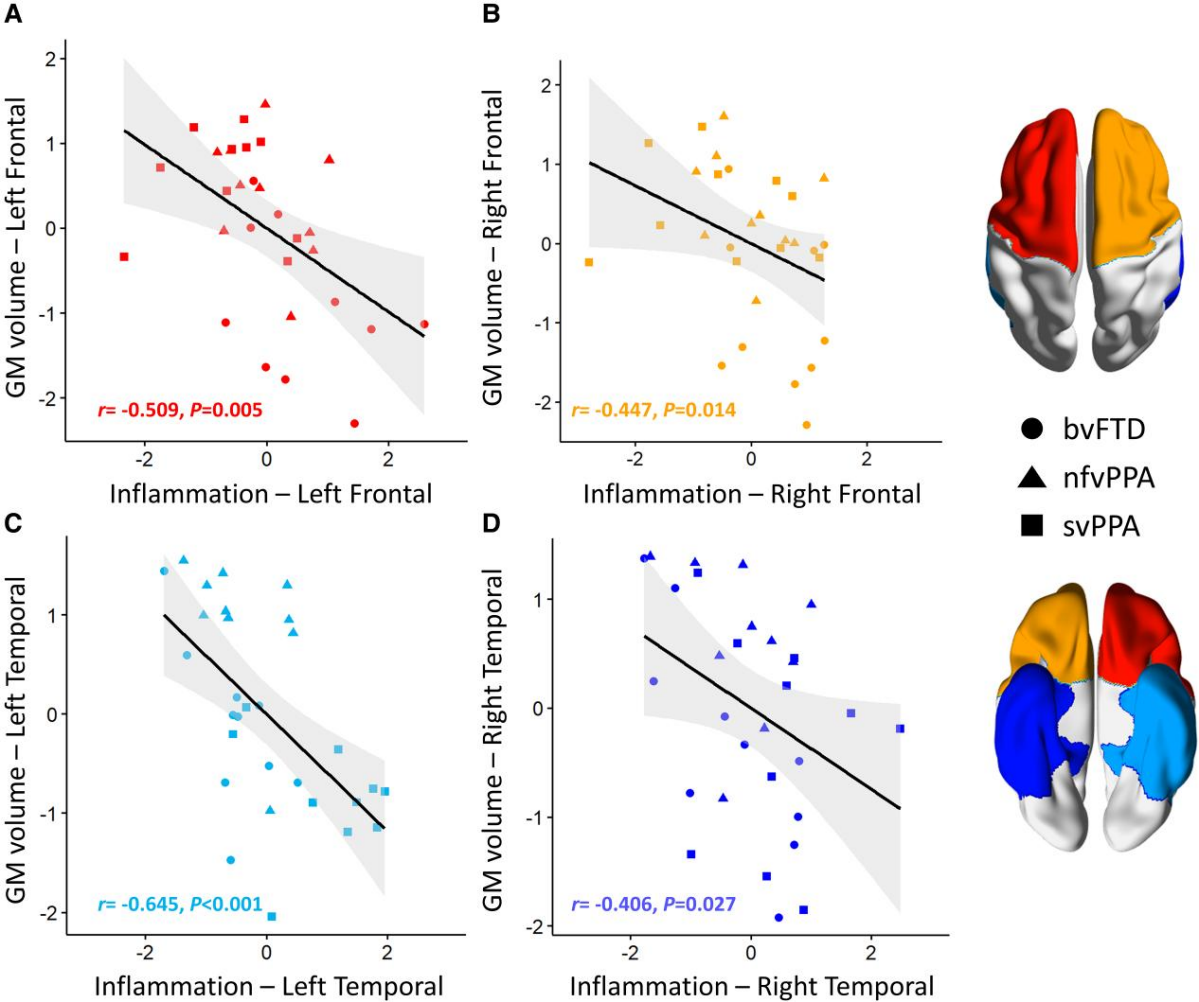
**Association between microglial activation and atrophy in frontal and temporal lobes.** Microglial activation (inflammation) is represented by [^11^C]PK11195 BP_ND_*z*-values, with atrophy by grey-matter (GM) volume *z*-values. Colour coding of plots refers to the four regions shown on the atlas brain axial view (*right*), whereas shapes represent clinical diagnostic groups. Coefficients and *P*-values were obtained from inter-modality Spearman's correlation analyses.

Between-modality correlations for lobar values of grey-matter volume and [^11^C]PK11195 BP_ND_ indicated significant negative associations in the left and right frontal lobes (left: *r* = −0.509, *P* = 0.005, p-FDR = 0.01; r*i*ght: *r* = −0.447, *P* = 0.014, p-FDR = 0.0187; Fig. 2A and B), and in the left and right temporal lobes (left: *r* = −0.645, *P* < 0.001, p-FDR = 0.004; right: *r* = −0.406, *P* = 0.027, p-FDR = 0.027, Fig. 2C and D). No significant correlations were found in the parietal and occipital lobes (Supplementary Fig. 2).

**Figure 3:**
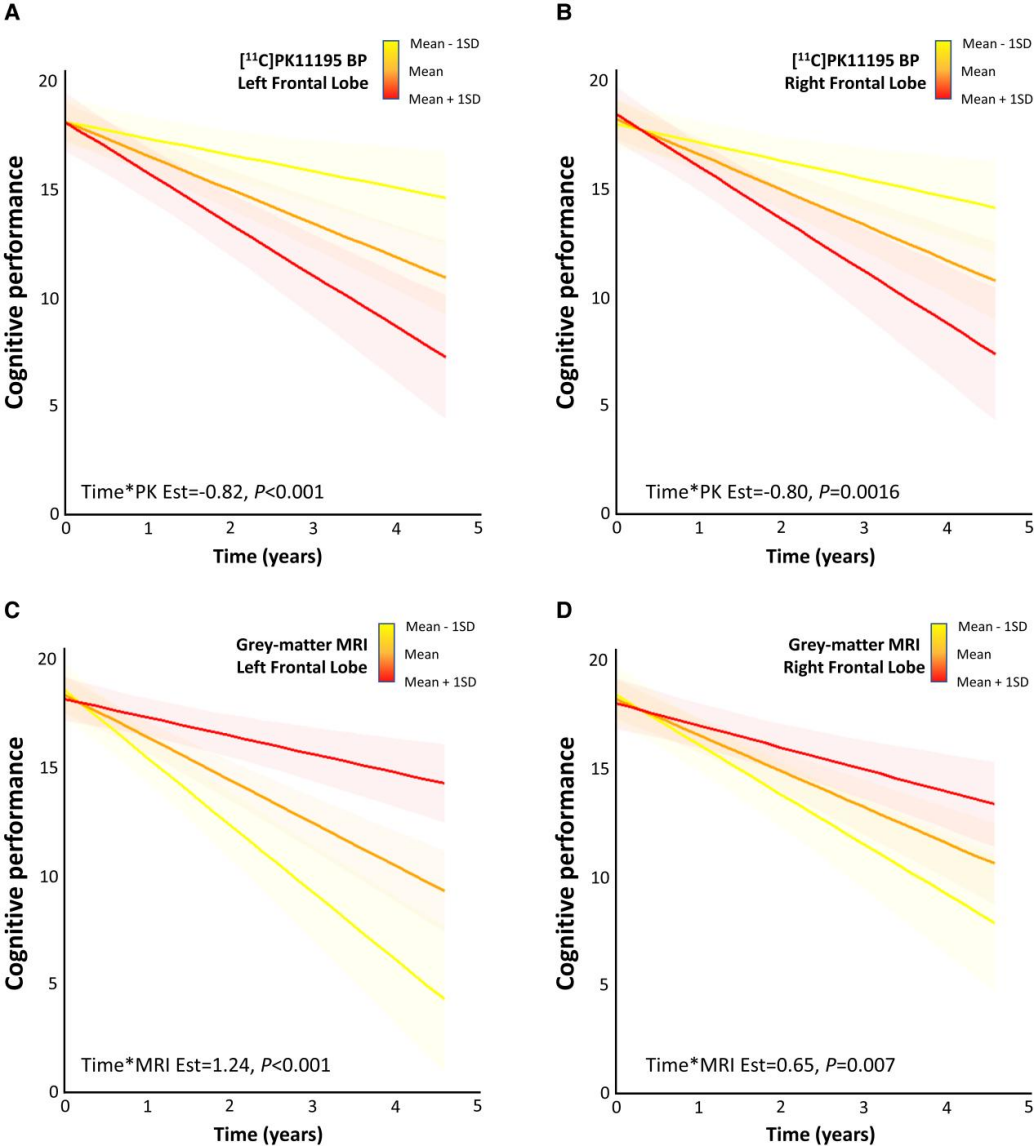
**Prognostic value of imaging measures in frontal regions.** (**A** and **B**) The interaction effects between baseline levels of microglial activation and time on cognitive performance (orange = average [^11^C]PK11195 BP_ND_ across all patients, yellow = mean − 1SD; red = mean + 1SD). (**C** and **D**) The interaction effects between baseline grey-matter (GM) volume and time on cognitive performance (orange = average GM volume, yellow = mean − 1SD; red = mean + 1SD). Estimates and *P*-values were obtained from one-step prediction linear mixed-effects models on ACE-R attention longitudinal scores and with imaging as predictors (Supplementary Table 2).

Using longitudinal ACE-R attention score as the primary outcome variable, with time intervals and single-modality imaging regional values as predictors, we identified a positive interaction effect between time and baseline grey-matter volumes in the left frontal lobe (estimate = 1.24, SE = 0.27, *t*-value = 4.67, *P* < 0.0001, p-FDR < 0.0001; Fig. 3C) and right frontal lobe (estimate = 0.65, SE = 0.23, *t*-value = 2.77, *P* = 0.007, p-FDR = 0.014; Fig. 3D and Supplementary Table 3). No significant positive associations between cognitive performance and grey-matter volumes were found in temporal lobes. Significant negative interaction effects between time and [^11^C]PK11195 BP_ND_ were found in left (estimate = −0.82, SE = 0.24, *t*-value = −3.46, *P* < 0.001, p-FDR = 0.0017; Fig. 3A) and right (estimate = −0.80, SE = 0.25, *t*-value = −3.27, *P* = 0.0016, p-FDR = 0.0212; Fig. 3B and Supplementary Table 3) frontal regions. No significant negative associations between cognitive performance and [^11^C]PK11195 BP_ND_ were found in temporal lobes. In addition, no significant associations between cognitive performance and either microglial activation or grey-matter volumes in parietal and occipital lobes were found. These results indicate that higher levels of microglial activation and lower grey-matter volume in the frontal lobes are associated with a faster cognitive decline, and are region-specific.

The results suggest a coarse co-localization in frontal lobes for the negative effects of grey-matter volume loss and microglial activation on cognitive dysfunction over time. To understand the contributions of these variables, we performed two additional linear mixed-effects models on longitudinal cognitive scores, with interaction terms between time intervals, grey-matter volume and [^11^C]PK11195 BP_ND_ of the left frontal lobe and the right frontal lobe, separately. Cognitive scores at baseline, age and education were included as covariates. In the left frontal lobe model, the interaction terms between time and each imaging predictor were significant (time × grey-matter volume: estimate = 1.09, SE = 0.26, *t*-value = 4.25, *P* < 0.0001; time × inflammation: estimate = −0.79, SE = 0.22, *t*-value = −3.60, *P* = 0.0006; Supplementary Table 4), however, no interaction effects were found between grey-matter volume and [^11^C]PK11195 BP_ND_ (inflammation × grey-matter volume *P* > 0.05). This confirms that atrophy and microglial activation are independent predictors of cognitive decline and provide additive information. In the right frontal lobe model, the interaction term between time and imaging predictor was significant for [^11^C]PK11195 BP_ND_ only (time × inflammation: estimate = −0.70, SE = 0.25, *t*-value = −2.86, *P* = 0.0055; Supplementary Table 4), whereas no interaction effects were found between grey-matter volume and [^11^C]PK11195 BP_ND_ (inflammation × grey-matter volume *P* > 0.05).

When an interaction term time × diagnosis was included in linear mixed-effect models with cognitive performance as dependent variable and frontal lobe single-modality imaging predictors (time × region of interest), a significant predictive effect of [^11^C]PK11195 BP_ND_ in the left frontal lobe was found (estimate = −0.70, SE = 0.27, *t*-value = −2.50, *P* = 0.01435, p-FDR = 0.0287; Supplementary Table 5), whereas interaction effects between time and grey-matter volume were not found (*P* > 0.05). Altogether, these results indicate that microglial activation and grey-matter volume are predictors across the FTDspectrum, but microglial activation severity in the left frontal lobe contributes information regardless of clinical variant.

Including an interaction term for time × gene in the linear mixed-effect models with cognitive performance as dependent variable and frontal lobe single-modality imaging predictors, we found a significant effect of genetic mutations, being associated with faster decline. However, the predictive effects of frontal grey-matter volume and microglial activation (interaction with time) remained significant in the models (Supplementary Table 6). Excluding the genetic cases from the linear mixed-effect analyses, did not change the main results on the predictive value of baseline grey-matter volumes and microglial activation in frontal regions (Supplementary Table 7).

Exploratory analyses with a one-step prediction modelling approach on [^11^C]PK11195 PET, including ACE-R subscores for other cognitive domains as outcome variables, identified only significant predictive effects of microglial activation in the right frontal lobe for scores on visuospatial subscale of ACE-R (estimate = −0.64, SE = 0.29, *t*-value = −2.23, *P* = 0.0285; Supplementary Table 8).

**Figure 4:**
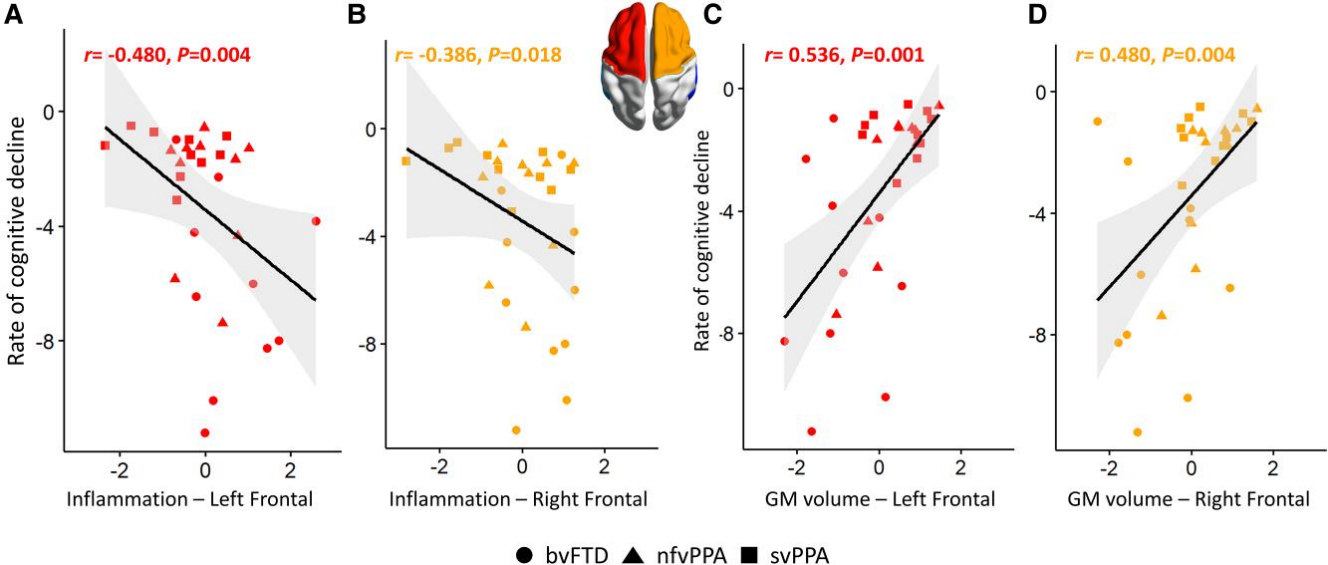
**Two-step confirmatory test of the prognostic value of imaging measures in frontal regions**. (**A** and **B**) The relationship between baseline levels of microglial activation in frontal regions (*x*-axis, [^11^C]PK11195 BP_ND_*z*-values) and annual rate of cognitive decline extracted from linear mixed-effects model on longitudinal cognitive scores (*y*-axis). (**C** and **D**) The relationship between baseline grey-matter (GM) volumes in frontal regions (*x*-axis, *z*-values) and annual rate of cognitive decline (*y*-axis). The shapes represent clinical diagnostic groups. Coefficients and *P*-values were obtained from inter-modality Spearman's correlation analyses.

The two-step procedure confirmed (i) negative associations of individual annual rate scores of cognitive decline (slope from linear mixed-effect model on longitudinal cognitive scores) with microglial activation levels in left (*r* = −0.480, *P* = 0.004, p-FDR = 0.016, BF_10_= 23.7; Fig. 4A) and right (*r* = −0.386, *P* = 0.018, p-FDR = 0.036, BF_10_= 4.2; Fig. 4B) frontal regions; and (ii) positive associations with grey-matter volumes in the left (*r* = 0.536, *P* = 0.001, p-FDR = 0.005, BF_10_= 150.0; Fig. 4C) and right (*r* = 0.480, *P* = 0.004, p-FDR = 0.008, BF_10_ = 18.4; Fig. 4D) frontal regions. Temporal regions did not show significant associations (Supplementary Fig. 3).

**Figure 5:**
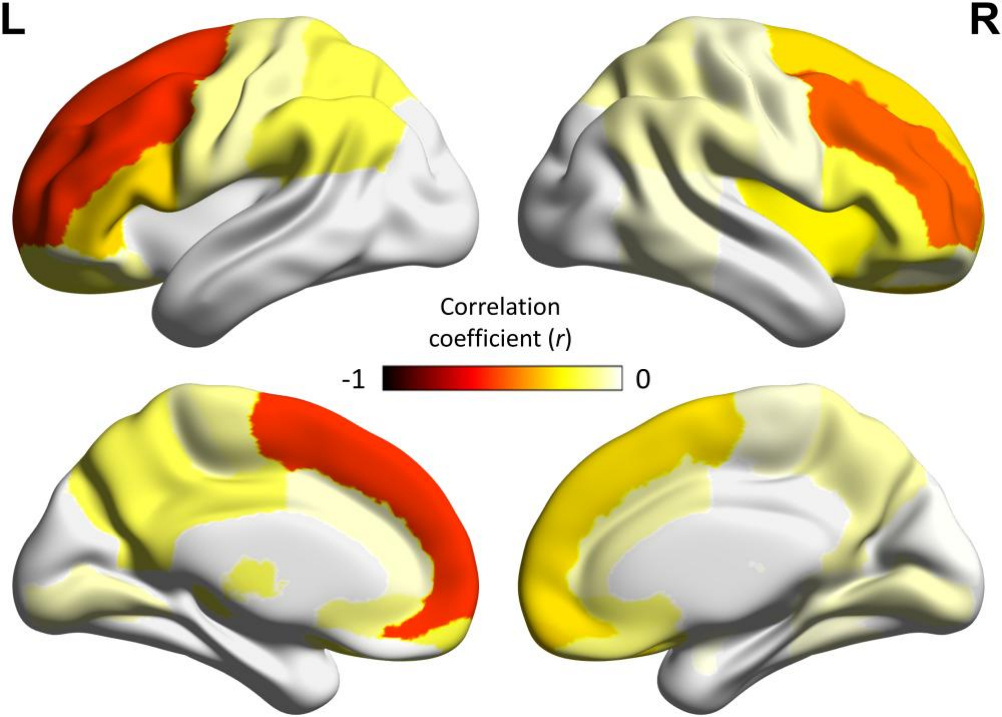
**Regional predictive effects of microglial activation.** Negative correlation coefficients from explorative regional analyses between annual rate of cognitive decline and regional [^11^C]PK11195 BP_ND_ within left (L) and right (R) frontal lobes.

Exploratory correlation analyses were performed between regional [^11^C]PK11195 BP_ND_ across the whole brain and annual rate scores of cognitive decline. Significant negative correlations were found with middle (left *r* = −0.558, right *r* = −0.483) and superior frontal gyri (left *r* = −0.551), the left inferior frontal gyrus (*r* = −0.307) and the right medial orbitofrontal cortex (*r* = −0.309, *P* < 0.05; Fig. 5).

## Discussion

This study demonstrates the complementary value of neuroimaging markers for microglial activation ([^11^C]PK11195 PET) and grey-matter volume (structural MRI) in predicting cognitive decline in three major variants of FTD. Higher levels of activated microglia at baseline in bilateral frontal cortex were associated with a faster deterioration in cognitive performance over 4 years, even when correcting for the clinical subtype of FTD. Grey-matter atrophy in frontal lobe was predictive of faster cognitive decline, when included in the same model as with frontal [^11^C]PK11195 PET signal. However, the predictive value of grey-matter volumes weakened, if correcting for clinical diagnosis. Overall, our results indicate that the PET measurement of microglial activation provides additional predictive information on cognitive decline across the FTDspectrum, over and above structural MRI, baseline cognitive performance and diagnosis.

For our study, we embraced a transdiagnostic approach to investigate the role of inflammation on cognitive decline across the FTDspectrum. Underling pathologies are likely to vary between bvFTD, nfvPPA and svPPA—with roughly 50% split between Tau and TDP43 pathologies in bvFTD, whereas predominant TDP43 and Tau pathology in svPPA and nfvPPA, respectively.^38^ However, our findings in living patients align with preclinical evidence showing that microglial activation has an active role in leading to synaptic damage and cognitive/motor impairments in animal models of both tauopathy^12,39^ and TDP-43 pathology.^13,40,41^ The coupling of inflammation and neuronal loss in FTD is also supported by post-mortem studies with human tissues, showing increased levels of activated microglia in frontotemporal regions in patients with a clinical diagnosis of FTD, and either tau or TDP-43 pathology.^6–8,15,18,42,43^ In our study, we included genetic cases, who may have different underlying pathophysiology. In a previous case series, we described symptom-related microglial activation patterns across the three main genetic mutations.^17^ Although genetic mutations were associated with faster decline than sporadic cases, predictive effects of frontal grey-matter volume and microglial activation remained significant when either including the genetic status as covariate in the models or excluding the genetic cases from the analyses. This suggests that genetic mutations did not drive the main results.

A separate issue is the regional anatomical variance of the three main FTD syndromes, which could lead to varying regional associations between inflammation and outcome. However, including diagnosis as a factor suggested the robustness of evidence for the prognostic value of brain inflammation across the frontotemporal spectrum, regardless of clinical variants and pathologies. Of note, when including clinical diagnosis in the model, microglial activation remained predictive of faster decline, especially on the left. This raises the question of asymmetry. svPPA is asymmetric, usually beginning/prominent on the left or on the right but in well-established disease it becomes bilateral. Similarly, nfvPPA is typically left dominant early on, but with progression it usually becomes bilateral. While bvFTD can be asymmetric, marked asymmetry is less common. Thus, the left sided PET associations here could be partly attributed to the asymmetry of pathology in PPA cases, and consequently the greater role of these regions in driving cognitive decline. Future PET-to-post-mortem studies across the spectrum may be able to identify pathology-specific inflammatory profiles.

In contrast to the widespread availability and use of structural MRI to study and diagnose FTD, *in vivo* imaging of inflammation has not been widely used. In patients with FTD, elevated microglial activation has been described in frontotemporal regions across all clinical variants by previous PET studies with TSPO ligands.^14–16,18^ However, these previous studies did not investigate the association between frontotemporal inflammation and progression. We had previously reported that in patients with genetic FTD, the distribution of microglial activation indexed by [^11^C]PK11195 PET reflects clinical heterogeneity and symptoms.^17^ However, longitudinal change from baseline presents a distinct challenge. Tracking cognition in FTD is complicated by the variability of neuropsychological changes between different clinical variants, such as relative episodic memory impairments in bvFTD^1^ and language deficits in primary progressive aphasia.^2^ However, progressive deficits in frontal and executive functions are observed across all three clinical syndromes,^44,45^ and in the presymptomatic phase of genetic FTD.^46–48^ Here, we report the significant association between *in vivo* microglial activation levels in frontal cortex and subsequent cognitive decline in patients with a clinical diagnosis of FTD, assessed as performance in attention/orientation tests. Despite microglial activation and grey-matter volumes in frontal regions individually correlating with cognitive decline over time, they did not interact in their association with annual rate of cognitive decline. This indicates additive and at least partially independent effects of activated microglia and atrophy on cognitive decline, although larger longitudinal studies will be needed to clarify their mechanistic relationships. Exploratory analyses of microglial prediction of other cognitive domains identified a significant association between baseline inflammation levels in the right frontal lobe and faster decline in visuospatial performance. This could reflect the progressive decline in other cognitive factors rather than core visuospatial impairments, such as attention and planning difficulties, but also apathy, impulsivity and disinhibition, as previously described.^49^

Our results on the prognostic value of regional microglial activation levels across the FTD spectrum align with similar evidence in patients with Alzheimer's disease^50^ and progressive supranuclear palsy,^51^ showing that region-specific between higher levels of microglial activation at baseline predict faster cognitive decline over time. In Alzheimer's disease, there have been many studies investigating microglial activation with TSPO PET and clinical severity (see^52^ for a recent review). Early and often small studies described null or even positive associations between TSPO PET signal and cognition—in particular, in patients with mild cognitive impairment. However, the weight of evidence over more recent and larger studies is in favour of the detrimental association of inflammation and progression. In particular, longitudinal imaging studies showed that TSPO tracer binding increases are generally associated with disease worsening,^53–55^ supporting cross-sectional studies and meta-analysis showing that higher cognitive deficits are associated with higher ligand binding.^50,56–59^ Across the Alzheimer's spectrum, microglial activation measured with TSPO PET at baseline can also be predictive of longitudinal tau propagation.^60^ These data together indicate that neuroinflammation in neurodegenerative diseases is not merely a pathological bystander, but actively participates in the cascade of events that defines individual clinical severity and prognosis in these patients. This supports the development of immunomodulatory strategies for disease-modifying treatments alone or in conjunction with treatments targeting other pathogenic processes. Thus, *in vivo* measures of neuroinflammation in patients with dementia may be valuable to predict their future clinical progression, and to classify them into fast or slow decliners—and to identify ideal time windows for usage of immunomodulatory drugs, which could empower patient stratification and target engagement in clinical trials.

Longitudinal studies in presymptomatic gene carriers may be particularly informative for the timeline and progression of pathological processes approaching dementia onset. A caveat to this approach is that mutations rarely cause svPPA. Previous PET studies with TSPO tracers have reported evidence that microglial activation may start early in FTD, preceding the development of the full syndrome in presymptomatic carriers of MAPT mutations.^61,62^ In particular, elevated levels of microglial activation were found in frontotemporal regions of a presymptomatic MAPT mutation carrier, compared to controls, despite a lack of protein aggregation and only marginal grey-matter atrophy, limited to the amygdala region.^61^ Levels of [^11^C]PK11195 binding in this presymptomatic carrier were comparable to those in a symptomatic patient with the same monogenetic mutation.^61^ Although longitudinal PET studies are needed to clarify the role of microglial activation as a promoter of junk protein accumulation or an early protective reaction, these results from presymptomatic FTD support the hypothesis that microglial activation may precede not only clinical symptom onset in FTD, but also the associated protein aggregation and neuronal loss.

Although [^11^C]PK11195 PET has been widely used to study microglial activation in neurodegenerative diseases, its application is not without limitations. The neuroinflammatory cascade associated with neurodegeneration is complex and, in addition to activated microglia, overexpressed TSPO can also be found in astrocytes and vascular smooth muscle cells.^63^ However, in the frontal cortex of patients with FTD, an autoradiography study on post-mortem tissues showed that [^11^C]PK11195 binding corresponds with activated microglia and correlates with immunohistochemistry evidence in the same region.^64^ [^11^C]PK11195 binds more strongly to activated microglia than quiescent microglia and reactive astrocytes.^65^ Another limitation of our study is the sample size. Our cohort is larger than most previous PET studies in FTD, however, the limited size of each variant-specific group does not enable the assessment between regional markers and specific cognitive domains within clinical diagnostic groups alone. This may be particularly problematic where atrophy is so severe as to reduce the signal-to-noise or precision of imaging markers, as can occur with temporal lobar change in svPPA. Owing to the limited sample size, we included age and education covariates only in the one-step prediction approach, as degrees of freedom are much reduced for the two-step approach. In addition, some cases declined rapidly and came back for follow-up visits only few weeks/months after baseline to complete a limited cognitive assessment (i.e. ACE-R). By using a linear mixed model of all data, we maximize the evidence for associations between imaging and outcome variables. In such a linear model, the short intervals are down-weighted proportionately in their contribution to the overall relationship. When we included only those cases and visits >1-year follow-up from baseline, 31 data points of cognitive scores were lost but the results remain essentially the same (Supplementary Table 9). Finally, our study does not assess longitudinal imaging changes, as patients underwent PET and MRI at the baseline visit and were followed up with cognitive assessments. We can assume that brain volumes and inflammation change with time, but this was out of the scope of our study.

In conclusion, our results indicate the relevance of *in vivo* microglial activation and grey-matter volume markers in frontal cortex to predict clinical progression in patients across the FTD spectrum. The combination of structural MRI and [^11^C]PK11195 PET to evaluate patients at baseline may be a valuable tool for clinical trials in FTD. Specifically, the combination of these pathological *in vivo* indices may be a useful marker to stratify patients with FTD into those likely to exhibit slow versus fast decline, improving cohort selection for clinical trials and the interpretation of clinical outcomes.

## Supplementary Material

awad078_Supplementary_Data
