# Supplementary material for: Microglial activation in the frontal cortex predicts cognitive decline in frontotemporal dementia
Source: Brain. 2023 Mar 8;146(8):3221–31. doi: 10.1093/brain/awad078 (PMC10393407; doi:10.1093/brain/awad078)
Supplement: awad078_Supplementary_Data [file awad078_supplementary_data.pdf]

Supplementary Material

Inflammation in the frontal cortex predicts cognitive decline in frontotemporal dementia

Maura Malpetti[1], Thomas E. Cope[1,2], Duncan Street[1], P. Simon Jones[1], Frank H. Hezemans[1,2], Elijah Mak[3], Kamen A. Tsvetanov[1], Timothy Rittman[1], W. Richard Bevan-Jones[3], Karalyn Patterson[1,2], Luca Passamonti[1], Tim D. Fryer[1,4], Young T. Hong[1,4], Franklin I. Aigbirhio[1,4], John T. O'Brien[3,*] and James B. Rowe[1,2,*]

1 Department of Clinical Neurosciences and Cambridge University Hospitals NHS Trust, University of Cambridge, Cambridge, United Kingdom
2 Medical Research Council Cognition and Brain Sciences Unit, Cambridge, United Kingdom
3 Department of Psychiatry, University of Cambridge, Cambridge, United Kingdom
4 Wolfson Brain Imaging Centre, University of Cambridge, Cambridge, United Kingdom
*Joint Senior authors

Corresponding Author:
Dr. Maura Malpetti
Department of Clinical Neurosciences
University of Cambridge
Herchel Smith Building, Forvie Site
Robinson Way, Cambridge Biomedical Campus
Cambridge CB2 0SZ
Email: mm2243@medschl.cam.ac.uk
Tel. no: +44 1223 760 696

Supplementary Table 1. Group comparisons of modality-specific regional z-score (Zsc) values across clinical phenotypes: behavioural variant frontotemporal dementia (bvFTD), non-fluent (nfvPPA) and semantic variants of primary progressive aphasia (svPPA). Significant differences between: bvFTD-nfvPPA *, bvFTD-svPPA †, nfvPPA-svPPA ^.

Modality	Region	bvFTD Zsc		nfvPPA Zsc		svPPA Zsc		Group Comparison		
		Mean	SD	Mean	SD	Mean	SD	F ANOVA	p	p FDR
	Temporal Lobe L	-0.11	0.80	0.93	0.71	-0.82	0.59	15.67 *^	0.000	0.001
	Temporal Lobe R	-0.31	1.02	0.62	0.71	-0.31	1.01	3.39	0.048	0.145
	Insula and Cingulate L	-0.67	0.97	0.84	0.69	-0.17	0.71	9.23 *^	0.001	0.004
	Insula and Cingulate R	-0.87	0.85	0.57	0.62	0.30	0.91	9.01 *^	0.001	0.004
	Occipital Lobe L	-0.11	1.06	0.09	1.19	0.02	0.81	0.09	0.914	0.945
	Occipital Lobe R	-0.08	1.31	-0.17	0.96	0.25	0.69	0.48	0.626	0.783
Grey-matter volumes (MRI)	Frontal Lobe L	-0.93	0.91	0.36	0.72	0.57	0.63	11.19 *†	0.000	0.003
	Frontal Lobe R	-0.89	1.02	0.44	0.67	0.45	0.64	9.48 *’	0.001	0.004
	Parietal Lobe L	-0.38	1.05	0.29	1.13	0.08	0.76	1.18	0.321	0.535
	Parietal Lobe R	-0.40	1.24	0.37	0.99	0.03	0.60	1.55	0.231	0.462
	Central Structures L	-0.85	0.71	0.56	0.78	0.29	0.92	8.59 *†	0.001	0.005
	Central Structures R	-0.88	0.70	0.60	0.67	0.28	0.97	9.56 *†	0.001	0.004
	Brainstem B	-0.35	1.14	0.09	1.01	0.26	0.82	0.98	0.387	0.580
	Cerebellum R	-0.11	1.24	-0.04	1.06	0.16	0.72	0.18	0.832	0.925
	Cerebellum L	-0.13	1.39	-0.02	0.94	0.15	0.59	0.19	0.825	0.925
	Temporal Lobe L	-0.54	0.63	-0.42	0.66	0.96	0.92	12.23 †	0.000	0.002
	Temporal Lobe R	-0.34	1.02	-0.14	0.79	0.49	1.07	2.00	0.155	0.358
	Insula and Cingulate L	0.19	0.83	-0.47	0.85	0.28	1.20	1.78	0.188	0.404
	Insula and Cingulate R	-0.05	1.09	-0.07	0.81	0.12	1.16	0.10	0.909	0.945
	Occipital Lobe L	-0.38	0.62	0.23	0.87	0.15	1.36	1.08	0.354	0.558
	Occipital Lobe R	-0.17	0.92	0.23	0.94	-0.07	1.18	0.40	0.671	0.805
	Frontal Lobe L	0.62	1.05	0.02	0.67	-0.64	0.89	5.14 †	0.013	0.043
C-PK11195 PET	Frontal Lobe R	0.49	0.75	0.00	0.72	-0.50	1.27	2.74	0.082	0.206
	Parietal Lobe L	0.11	1.43	0.19	0.67	-0.30	0.76	0.68	0.516	0.704
	Parietal Lobe R	0.13	1.14	0.29	0.73	-0.42	1.04	1.44	0.254	0.476
	Central Structures L	0.03	1.52	-0.03	0.53	0.00	0.79	0.01	0.992	0.992
	Central Structures R	0.35	1.14	-0.15	0.74	-0.20	1.09	0.92	0.411	0.588
	Brainstem B	-0.52	1.22	0.51	0.76	0.01	0.75	3.00	0.067	0.181
	Cerebellum R	-0.41	1.45	0.19	0.58	0.22	0.72	1.29	0.292	0.515
	Cerebellum L	-0.26	1.45	0.19	0.68	0.07	0.74	0.53	0.595	0.775

Supplementary Table 2. Sample sizes required for a 2-arm, 1-year follow-up therapeutic trial to detect 25%, 50%, and 75% changes using ACE-R attention sub-score as outcome measure. Individual estimates of cognitive decline from linear mixed effect model on ACE-R attention subscores were used to calculate a standardised effect size with the equation: "Effect size = (μ(Estimates) $*\ p$) / sd(Estimates)" - similar to previous reports (Sakpal, 2010; Staffaroni et al., 2019; Stamelou et al., 2016). The standardised effect size is therefore a product of the mean (μ) and standard deviation (sd) of the estimated rate of change of the variable of interest (ie ACE-R attention sub-score) and the percentage of expected change considered clinically relevant (p), for example 0.50 or 50%. Then we performed power calculations using this output in the pwr package (Champely et al., 2017) to determine the required sample size per group for a 2-sample t test (i.e. for a 2-arm, 1-year follow-up therapeutic trial) based on a 2-sided significance level of 5% and a power of 80%. A range of theoretical percentage changes were used (i.e. 25%, 50%, 75%). An approximation of the sample size adjusting for a drop-out rate of 25% was also presented using the formula "Estimated sample size / 0.75". It can be seen from the table that the estimated sample sizes are in a feasible range for realistic clinical trials lasting one year.

ACE-R Attention Estimate LMEM (mean, SD)	25% change			50% change			75% change		
	Effect size	Sample Size	Sample Size with 25% drop-out	Effect size	Sample Size	Sample Size with 25% drop-out	Effect size	Sample Size	Sample Size with 25% drop-out
-3.42 ± 3.06	-0.279	202	269	-0.559	51	68	-0.838	23	31

Supplementary Table 3. One-step prediction approach results. Significant linear mixed effect models longitudinal ACE-R attention score as the primary outcome variable, and time intervals and single-modality imaging regional values as predictors (top panel: grey-matter volumes, bottom panel: [11C]PK11195 PET as predictors). Note: Zsc is short for z-score.

**Outcome: ACE-R Att/Or**	**Left Frontal Lobe**			**Right Frontal Lobe**		
Predictors (MRI model)	*Estimates*	*95% CI*	*p*	*Estimates*	*95% CI*	*p*
(Intercept)	-2.56	-8.87 – 3.74	0.422	-2.97	-10.13 – 4.20	0.413
Time	-2.32	-2.88 – -1.75	**<0.001**	-1.81	-2.33 – -1.28	**<0.001**
Age	0	-0.07 – 0.07	0.976	0	-0.08 – 0.08	0.92
Education Years	0.03	-0.19 – 0.25	0.785	0.06	-0.18 – 0.30	0.636
ACER Att/Or Baseline	1.14	0.93 – 1.36	**<0.001**	1.15	0.92 – 1.37	**<0.001**
Frontal Lobe L GM Zsc	-0.22	-1.07 – 0.62	0.602			
**Time * Frontal Lobe L GM Zsc**	1.24	0.71 – 1.76	**<0.001**			
Frontal Lobe R GM Zsc				-0.2	-1.08 – 0.68	0.656
**Time * Frontal Lobe R GM Zsc**				0.65	0.18 – 1.11	**0.007**
Predictors (PET model)	*Estimates*	*95% CI*	*p*	*Estimates*	*95% CI*	*p*
(Intercept)	-1.85	-8.30 – 4.60	0.571	-1.89	-8.25 – 4.47	0.557
Time	-1.7	-2.15 – -1.24	**<0.001**	-1.74	-2.22 – -1.27	**<0.001**
Age	0	-0.07 – 0.08	0.922	-0.01	-0.08 – 0.07	0.83
Education Years	-0.05	-0.32 – 0.22	0.731	-0.03	-0.28 – 0.22	0.831
ACER Att/Or Baseline	1.13	0.92 – 1.34	**<0.001**	1.17	0.96 – 1.38	**<0.001**
Frontal Lobe L PK Zsc	0	-0.88 – 0.88	0.997			
**Time * Frontal Lobe L PK Zsc**	-0.82	-1.30 – -0.35	**0.001**			
Frontal Lobe R PK Zsc				0.24	-0.55 – 1.03	0.545
**Time * Frontal Lobe R PK Zsc**				-0.8	-1.29 – -0.31	**0.002**

Supplementary Table 4. One-step prediction results with between-modalities interaction terms. Significant linear mixed effect models longitudinal ACE-R attention score as the outcome variable, and interaction terms between time intervals, grey-matter volume and [11C]PK11195 PET in frontal regions. Note: Zsc is short for z-score.

**Outcome: ACE-R Att/Or**	**Left Frontal Lobe**			**Right Frontal Lobe**		
Predictors	*Estimates*	*95% CI*	*p*	*Estimates*	*95% CI*	*p*
(Intercept)	-1.3	-7.12 – 4.53	0.659	-1.41	-8.75 – 5.92	0.703
Time	-2.38	-3.00 – -1.77	**<0.001**	-2.39	-3.02 – -1.77	**<0.001**
Age	-0.01	-0.07 – 0.06	0.858	-0.01	-0.10 – 0.07	0.725
Education Years	-0.04	-0.26 – 0.19	0.746	-0.01	-0.26 – 0.24	0.939
ACER Att/Or Baseline	1.15	0.95 – 1.34	**<0.001**	1.17	0.95 – 1.39	**<0.001**
Frontal Lobe L PK Zsc	0.27	-0.56 – 1.10	0.521			
Frontal Lobe L GM Zsc	-0.2	-1.00 – 0.60	0.617			
**Time * Frontal Lobe L PK Zsc**	-0.79	-1.22 – -0.35	**0.001**			
**Time * Frontal Lobe L GM Zsc**	1.09	0.58 – 1.60	**<0.001**			
Frontal Lobe L PK Zsc * Frontal Lobe L GM Zsc	0.16	-0.62 – 0.93	0.69			
Time * Frontal Lobe L PK Zsc * Frontal Lobe L GM Zsc	0.35	-0.19 – 0.90	0.202			
Frontal Lobe R PK Zsc				0.23	-0.56 – 1.02	0.569
Frontal Lobe R GM Zsc				-0.12	-0.99 – 0.75	0.786
**Time * Frontal Lobe R PK Zsc**				-0.7	-1.19 – -0.21	**0.005**
**Time * Frontal Lobe R GM Zsc**				0.45	-0.01 – 0.91	0.056
Frontal Lobe R PK Zsc * Frontal Lobe R GM Zsc				0.63	-0.32 – 1.59	0.191
Time * Frontal Lobe R PK Zsc * Frontal Lobe R GM Zsc				-0.76	-1.45 – -0.07	0.031

Supplementary Table 5. One-step prediction results with time*diagnosis interaction term. Significant linear mixed effect models longitudinal ACE-R attention score as the outcome variable, and interaction terms between time intervals and [11C]PK11195 PET in frontal regions, time intervals and diagnosis. Note: Zsc is short for z-score.

**Outcome: ACE-R Att/Or**	**Left Frontal Lobe**			**Right Frontal Lobe**		
Predictors (PET model)	*Estimates*	*95% CI*	*p*	*Estimates*	*95% CI*	*p*
(Intercept)	-0.27	-5.95 – 5.40	0.924	0	-5.76 – 5.77	1
Time	-3.69	-4.77 – -2.60	**<0.001**	-3.73	-4.85 – -2.61	**<0.001**
EntryAge	-0.03	-0.10 – 0.05	0.476	-0.03	-0.10 – 0.04	0.345
Education Years	0.02	-0.22 – 0.27	0.855	0.03	-0.20 – 0.26	0.823
Bas ACE R Attention	1.08	0.88 – 1.28	**<0.001**	1.08	0.88 – 1.29	**<0.001**
Diagnosis (nfvPPA)	0.55	-1.50 – 2.59	0.596	0.69	-1.26 – 2.65	0.482
Diagnosis (svPPA)	0.75	-1.34 – 2.85	0.477	0.73	-1.31 – 2.77	0.478
Time * Diagnosis (nfvPPA)	2.6	1.38 – 3.82	**<0.001**	2.59	1.31 – 3.88	**<0.001**
Time * Diagnosis (svPPA)	2.04	0.64 – 3.43	**0.005**	2.25	0.75 – 3.74	**0.004**
Frontal Lobe L PK Zsc	0.34	-0.48 – 1.16	0.408			
**Time * Frontal Lobe L PK Zsc**	-0.67	-1.20 – -0.14	**0.014**			
Frontal Lobe R PK Zsc				0.36	-0.33 – 1.06	0.301
**Time * Frontal Lobe R PK Zsc**				-0.48	-1.04 – 0.09	0.096

Supplementary Table 6. One-step prediction results with time*gene interaction term. Significant linear mixed effect models longitudinal ACE-R attention score as the outcome variable, and interaction terms between time intervals, grey-matter volume and [11C]PK11195 PET in frontal regions, and between time intervals and genetic status (1 = gene mutation, 0 = sporadic/unknown gene cases). Note: Zsc is short for z-score.

**Outcome: ACE-R Att/Or**	**Left Frontal Lobe**			**Right Frontal Lobe**		
Predictors (MRI model)	*Estimates*	*95% CI*	*p*	*Estimates*	*95% CI*	*p*
(Intercept)	0.07	-8.74 – 8.89	0.987	-0.76	-<br>10.69 – 9.17	0.879
Time	-2.04	-2.62 – -1.47	**<0.001**	-1.56	-2.08 – -1.04	**<0.001**
Age	-0.02	-0.09 – 0.06	0.676	-0.02	-0.11 – 0.07	0.705
Gene	0.19	-2.17 – 2.55	0.872	0.5	-2.09 – 3.08	0.704
Education Years	-0.03	-0.26 – 0.19	0.768	-0.01	-0.25 – 0.23	0.939
ACER Att/Or Baseline	1.09	0.82 – 1.36	**<0.001**	1.12	0.83 – 1.41	**<0.001**
**Time * Gene**	-3.42	-5.74 – -1.11	**0.004**	-4.13	-6.54 – -1.73	**0.001**
Frontal Lobe L GM Zsc	-0.04	-0.87 – 0.80	0.932			
**Time * Frontal Lobe L GM Zsc**	0.98	0.45 – 1.52	**<0.001**			
Frontal Lobe R GM Zsc				-0.06	-0.92 – 0.80	0.893
**Time * Frontal Lobe R GM Zsc**				0.44	-0.02 – 0.90	0.058
Predictors (PET model)	*Estimates*	*95% CI*	*p*	*Estimates*	*95% CI*	*p*
(Intercept)	0.64	-8.32 – 9.59	0.888	0.29	-8.82 – 9.39	0.95
Time	-1.53	-1.97 – -1.10	**<0.001**	-1.57	-2.03 – -1.11	**<0.001**
Age	-0.01	-0.09 – 0.07	0.791	-0.02	-0.10 – 0.06	0.609
Gene	0.39	-2.07 – 2.85	0.754	0.43	-2.10 – 2.96	0.738
Education Years	-0.12	-0.38 – 0.14	0.365	-0.09	-0.33 – 0.16	0.484
ACER Att/Or Baseline	1.09	0.83 – 1.36	**<0.001**	1.14	0.87 – 1.40	**<0.001**
**Time * Gene**	-4.22	-6.47 – -1.97	**<0.001**	-4.23	-6.53 – -1.93	**<0.001**
Frontal Lobe L PK Zsc	-0.14	-0.97 – 0.70	0.747			
**Time * Frontal Lobe L PK Zsc**	-0.69	-1.14 – -0.24	**0.003**			
Frontal Lobe R PK Zsc				0.13	-0.63 – 0.90	0.727
**Time * Frontal Lobe R PK Zsc**				-0.66	-1.13 – -0.20	**0.006**

Supplementary Table 7. One-step prediction approach results excluding genetic cases. Significant linear mixed effect models longitudinal ACE-R attention score as the primary outcome variable, and time intervals and single-modality imaging regional values as predictors (top panel: grey-matter volumes, bottom panel: [11C]PK11195 PET as predictors). Note: Zsc is short for z-score.

**Outcome: ACE-R Att/Or**	**Left Frontal Lobe**			**Right Frontal Lobe**		
Predictors (MRI model)	*Estimates*	*95% CI*	*p*	*Estimates*	*95% CI*	*p*
(Intercept)	-0.94	-10.85 – 8.96	0.85	-1.24	-12.19 – 9.70	0.821
Time	-2.07	-2.64 – -1.49	**<0.001**	-1.59	-2.11 – -1.06	**<0.001**
Age	-0.02	-0.10 – 0.07	0.685	-0.03	-0.13 – 0.07	0.551
Education Years	0.01	-0.23 – 0.25	0.933	0.04	-0.23 – 0.31	0.757
ACER Att/Or Baseline	1.12	0.74 – 1.50	**<0.001**	1.15	0.76 – 1.54	**<0.001**
Frontal Lobe L GM Zsc	0.22	-0.76 – 1.20	0.655			
**Time * Frontal Lobe L GM Zsc**	0.98	0.44 – 1.52	**0.001**			
Frontal Lobe R GM Zsc				0.3	-0.70 – 1.30	0.547
Time * Frontal Lobe R GM Zsc				0.42	-0.05 – 0.88	0.081
Predictors (PET model)	*Estimates*	*95% CI*	*p*	*Estimates*	*95% CI*	*p*
(Intercept)	-1.84	-11.55 – 7.87	0.707	-2.24	-12.18 – 7.71	0.656
Time	-1.56	-2.00 – -1.12	**<0.001**	-1.61	-2.07 – -1.14	**<0.001**
Age	0	-0.09 – 0.09	0.987	-0.02	-0.11 – 0.07	0.663
Education Years	-0.11	-0.42 – 0.20	0.48	-0.06	-0.36 – 0.24	0.698
ACER Att/Or Baseline	1.19	0.84 – 1.55	**<0.001**	1.26	0.91 – 1.60	**<0.001**
Frontal Lobe L PK Zsc	-0.24	-1.21 – 0.73	0.625			
**Time * Frontal Lobe L PK Zsc**	-0.7	-1.16 – -0.24	**0.003**			
Frontal Lobe R PK Zsc				0.13	-0.72 – 0.99	0.758
**Time * Frontal Lobe R PK Zsc**				-0.68	-1.16 – -0.21	**0.005**

Supplementary Table 8. Explorative analyses of inflammation predictive effect for other cognitive domains. Significant linear mixed effect models longitudinal ACE-R visuospatial score as the primary outcome variable, and time intervals and [11C]PK11195 PET in frontal regions. Note: Zsc is short for z-score.

**Outcome: ACE-R Visuosp**	**Left Frontal Lobe**			**Right Frontal Lobe**		
Predictors (PET model)	*Estimates*	*95% CI*	*p*	*Estimates*	*95% CI*	*p*
(Intercept)	3.67	-4.82 – 12.15	0.393	2.77	-5.46 – 11.00	0.506
Time	-1.1	-1.63 – -0.57	**<0.001**	-1.22	-1.77 – -0.67	**<0.001**
Age	-0.05	-0.14 – 0.03	0.24	-0.06	-0.15 – 0.03	0.174
Education Years	0	-0.35 – 0.35	0.998	0.01	-0.33 – 0.35	0.949
ACER Att/Or Baseline	0.95	0.49 – 1.42	**<0.001**	1.05	0.59 – 1.51	**<0.001**
Frontal Lobe L PK Zsc	-0.41	-1.42 – 0.59	0.416			
Time * Frontal Lobe L PK Zsc	-0.46	-1.03 – 0.10	0.106			
Frontal Lobe R PK Zsc				-0.02	-0.91 – 0.88	0.972
Time * Frontal Lobe R PK Zsc				-0.64	-1.20 – -0.07	**0.028**

Supplementary Table 9. One-step prediction approach results excluding follow-up visits < 1 year from baseline. Significant linear mixed effect models longitudinal ACE-R attention score as the primary outcome variable, and time intervals and single-modality imaging regional values as predictors (top panel: grey-matter volumes, bottom panel: [11C]PK11195 PET as predictors). Note: Zsc is short for z-score.

**Outcome: ACE-R Att/Or**	**Left Frontal Lobe**			**Right Frontal Lobe**		
Predictors (MRI model)	*Estimates*	*95% CI*	*p*	*Estimates*	*95% CI*	*p*
(Intercept)	-0.68	-7.00 – 5.64	0.829	-0.38	-7.56 – 6.80	0.916
Time	-2.29	-2.85 – -1.72	**<0.001**	-1.81	-2.35 – -1.27	**<0.001**
Age	-0.04	-0.11 – 0.03	0.267	-0.04	-0.12 – 0.03	0.259
Education Years	0.05	-0.17 – 0.26	0.669	0.06	-0.17 – 0.29	0.614
ACER Att/Or Baseline	1.17	0.96 – 1.39	**<0.001**	1.17	0.94 – 1.39	**<0.001**
Frontal Lobe L GM Zsc	-0.29	-1.12 – 0.54	0.485			
**Time * Frontal Lobe L GM Zsc**	1.16	0.64 – 1.68	**<0.001**			
Frontal Lobe R GM Zsc				-0.25	-1.12 – 0.63	0.577
**Time * Frontal Lobe R GM Zsc**				0.62	0.15 – 1.09	**0.011**
Predictors (PET model)	*Estimates*	*95% CI*	*p*	*Estimates*	*95% CI*	*p*
(Intercept)	0.12	-6.41 – 6.65	0.971	0.94	-5.43 – 7.31	0.769
Time	-1.64	-2.10 – -1.18	**<0.001**	-1.71	-2.19 – -1.23	**<0.001**
Age	-0.04	-0.11 – 0.04	0.329	-0.05	-0.13 – 0.02	0.161
Education Years	0	-0.27 – 0.26	0.983	-0.03	-0.27 – 0.22	0.818
ACER Att/Or Baseline	1.15	0.93 – 1.36	**<0.001**	1.18	0.98 – 1.39	**<0.001**
Frontal Lobe L PK Zsc	0.18	-0.69 – 1.05	0.675			
**Time * Frontal Lobe L PK Zsc**	-0.71	-1.20 – -0.23	**0.005**			
Frontal Lobe R PK Zsc				0.31	-0.47 – 1.09	0.429
**Time * Frontal Lobe R PK Zsc**				-0.76	-1.25 – -0.26	**0.003**

Supplementary Figure 1. Decline over time in other cognitive domains. Cognitive performance measured by revised Addenbrooke's Cognitive Examination (ACE-R), with sub-scores for fluency (A), language (B), memory (C), visuospatial (D) domains. Dashed lines represent raw scores, bold coloured lines chart the time-course for individual patients estimated by the domain-specific linear mixed effect models, while the black line represents the linear estimated change at the group level (bvFTD = behavioural variant frontotemporal dementia, nfvPPA = non-fluent variant primary progressive aphasia, svPPA = semantic variant primary progressive aphasia). Significant decline at group level was identified for all cognitive domains, but for the fluency and memory sub-scores, the random slope was not significant (no inter-subject variability).

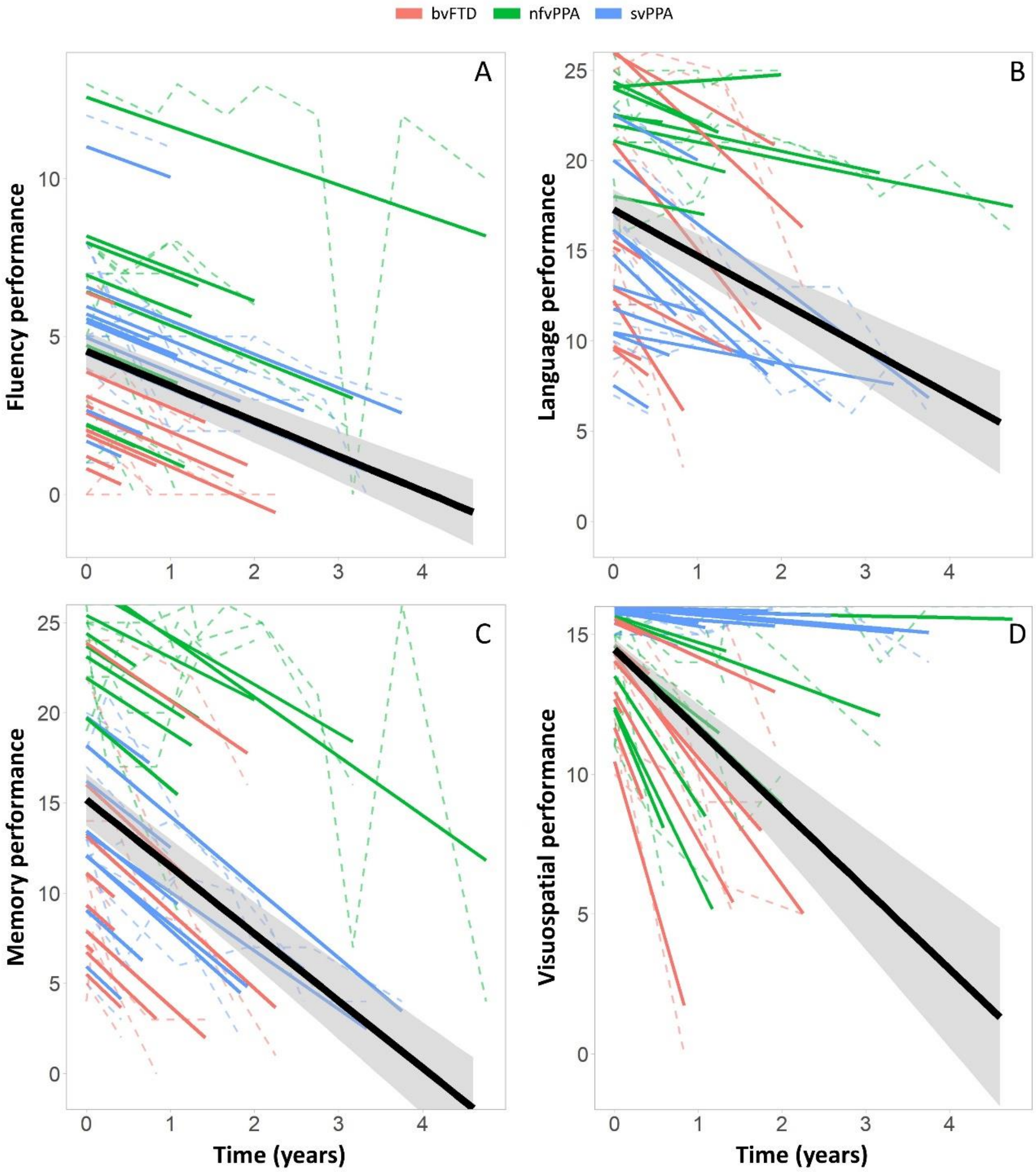

Supplementary Figure 2. Association between microglial activation and atrophy in parietal and occipital lobes. Microglial activation (inflammation) is represented by [^{11}C]PK11195 binding potential (BP_{ND}) z-values, with atrophy by grey-matter (GM) volume z-values.

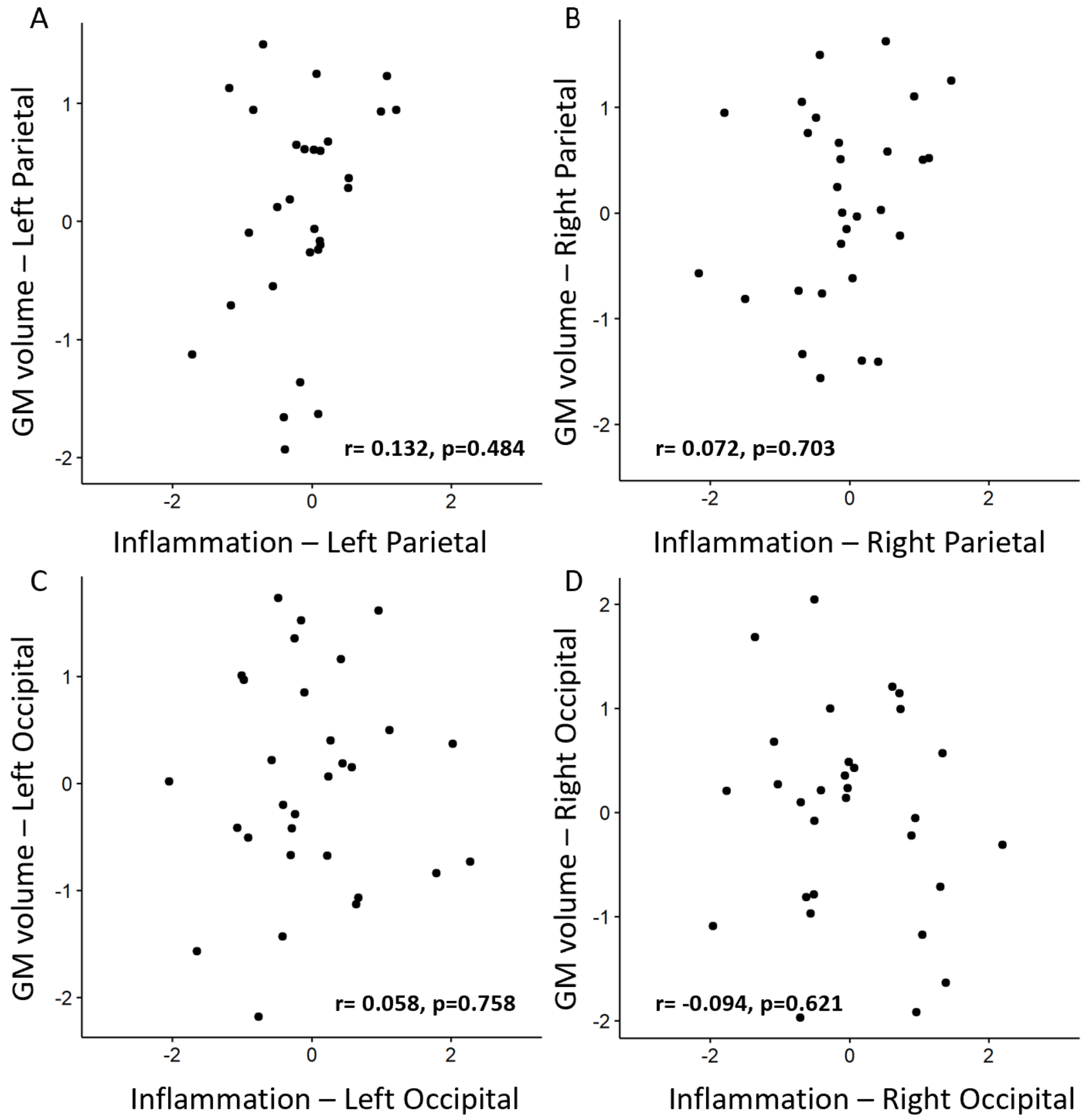

Supplementary Figure 3. Two-step confirmatory test of the prognostic value of imaging measures in temporal regions. Panels A and B: relationship between baseline levels of microglial activation in temporal regions (x-axis, [^{11}C]PK11195 binding potential (BP_{ND}) z-values) and annual rate of cognitive decline extracted from linear mixed-effect model on longitudinal cognitive scores (y-axis). Panels C and D: relationship between baseline grey-matter volumes in temporal regions (x-axis, z-values) and annual rate of cognitive decline (y-axis).

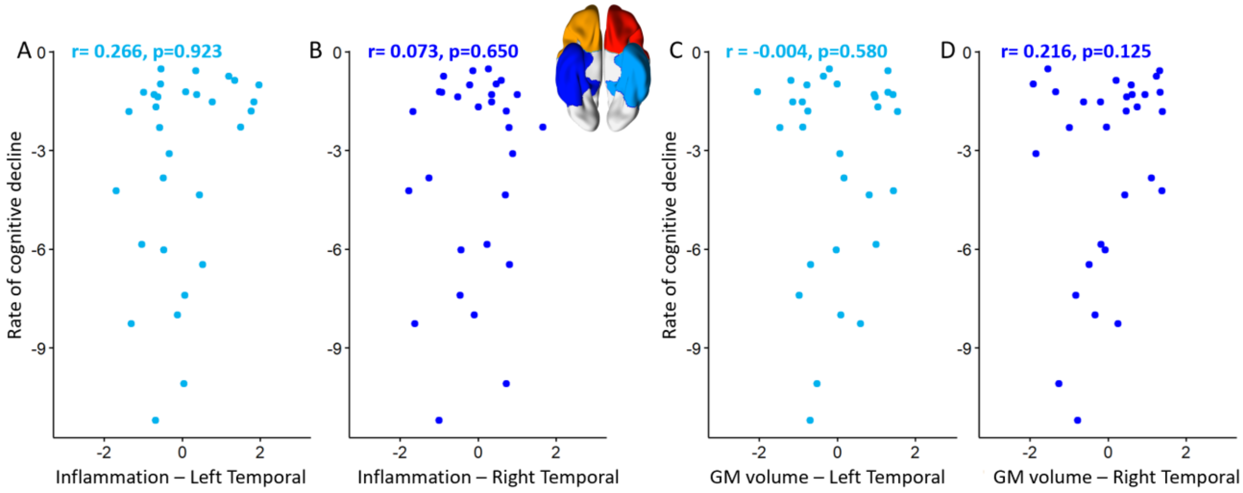

Supplementary References

Champely, S., Ekstrom, C., Dalgaard, P., Gill, J., Weibelzahl, S., & Anandkumar, A. (2017). pwr: Basic Functions for Power Analysis. https://cran.rproject.org/web/packages/pwr/index.html

Sakpal, T. V. (2010). Sample size estimation in clinical trial. Perspectives in Clinical Research, 1(2), 67–69. http://www.ncbi.nlm.nih.gov/pubmed/21829786

Staffaroni, A. M., Ljubenkov, P. A., Kornak, J., Cobigo, Y., Datta, S., Marx, G., Walters, S. M., Chiang, K., Olney, N., Elahi, F. M., Knopman, D. S., Dickerson, B. C., Boeve, B. F., Gorno-Tempini, M. L., Spina, S., Grinberg, L. T., Seeley, W. W., Miller, B. L., Kramer, J. H., … Rosen, H. J. (2019). Longitudinal multimodal imaging and clinical endpoints for frontotemporal dementia clinical trials. Brain : A Journal of Neurology, 142(2), 443–459. https://doi.org/10.1093/brain/awy319

Stamelou, M., & Höglinger, G. (2016). A Review of Treatment Options for Progressive Supranuclear Palsy. CNS Drugs, 30(7), 629–636. https://doi.org/10.1007/s40263-016-0347-2
